# STAT6 Deficiency Attenuates Myeloid Fibroblast Activation and Macrophage Polarization in Experimental Folic Acid Nephropathy

**DOI:** 10.3390/cells10113057

**Published:** 2021-11-06

**Authors:** Baihai Jiao, Changlong An, Hao Du, Melanie Tran, Penghua Wang, Dong Zhou, Yanlin Wang

**Affiliations:** 1 Division of Nephrology, Department of Medicine, University of Connecticut School of Medicine, Farmington, CT 06030, USA; bjaio@uchc.edu (B.J.); an@uchc.edu (C.A.); hdu@uchc.edu (H.D.); metran@uchc.edu (M.T.); dzhou@uchc.edu (D.Z.); 2 Department of Immunology, University of Connecticut School of Medicine, Farmington, CT 06030, USA; pewang@uchc.edu; 3 Department of Cell Biology, University of Connecticut School of Medicine, Farmington, CT 06030, USA; 4 Institute for Systems Genomics, University of Connecticut School of Medicine, Farmington, CT 06030, USA; 5 Renal Section, Veterans Affairs Connecticut Healthcare System, West Haven, CT 06516, USA

**Keywords:** STAT6, macrophage polarization, fibroblasts, fibrosis, chronic kidney disease

## Abstract

Renal fibrosis is a pathologic feature of chronic kidney disease, which can lead to end-stage kidney disease. Myeloid fibroblasts play a central role in the pathogenesis of renal fibrosis. However, the molecular mechanisms pertaining to myeloid fibroblast activation remain to be elucidated. In the present study, we examine the role of signal transducer and activator of transcription 6 (STAT6) in myeloid fibroblast activation, macrophage polarization, and renal fibrosis development in a mouse model of folic acid nephropathy. STAT6 is activated in the kidney with folic acid nephropathy. Compared with folic-acid-treated wild-type mice, STAT6 knockout mice had markedly reduced myeloid fibroblasts and myofibroblasts in the kidney with folic acid nephropathy. Furthermore, STAT6 knockout mice exhibited significantly less CD206 and PDGFR-β dual-positive fibroblast accumulation and M2 macrophage polarization in the kidney with folic acid nephropathy. Consistent with these findings, STAT6 knockout mice produced less extracellular matrix protein, exhibited less severe interstitial fibrosis, and preserved kidney function in folic acid nephropathy. Taken together, these results have shown that STAT6 plays a critical role in myeloid fibroblasts activation, M2 macrophage polarization, extracellular matrix protein production, and renal fibrosis development in folic acid nephropathy. Therefore, targeting STAT6 may provide a novel therapeutic strategy for fibrotic kidney disease.

## 1. Introduction

Chronic kidney disease (CKD) is a concerning public health issue that affects more than 10% of the world’s population [1,2]. Renal fibrosis, a common pathological hallmark of CKD, is the inevitable consequence of fibroblast activation and the production and deposition of the extracellular matrix (ECM) [3]. There are no effective therapeutic strategies to halt and/or reverse the progression of fibrosis. The molecular mechanisms leading to renal fibrosis are incompletely understood; hence, a greater knowledge of the pathophysiological mechanisms is imperative to discover new therapeutic strategies for CKD.

Recent studies have indicated that bone-marrow-derived fibroblasts, termed fibrocytes, make an important contribution to the populations of activated fibroblasts and have a crucial role in renal fibrosis development [4,5,6,7,8,9]. Bone-marrow-derived fibroblasts share the characteristics of fibroblasts, such as the expression of collagen I, platelet-derived growth factor receptor-β (PDGFR-β), and vimentin, as well as the characteristics of hematopoietic cells, such as the expression of CD45 and CD11b [10,11]. Our previous studies have demonstrated that these cells contribute significantly to the pathogenesis of renal fibrosis [12,13,14]. Recent studies showed that STAT6 participates in the pathogenesis of lung [15,16], renal [12,17,18], and liver fibrosis [19,20].

In the present study, we used STAT6 knockout (KO) mice to investigate the role of STAT6 in bone-marrow-derived fibroblast activation, macrophage polarization, and the development of renal fibrosis in folic acid nephropathy. Our results demonstrate that STAT6 deletion in mice reduces the accumulation of myeloid fibroblasts and myofibroblasts, inhibits M2 macrophage polarization, and decreases the production of extracellular matrix proteins in the kidney, thereby attenuating the development of renal fibrosis and preserving renal function in folic-acid-induced nephropathy.

## 2. Materials and Methods

### 2.1. Experimental Animals

Age-matched male wild-type (WT) and STAT6 knockout (KO) mice [21] (8 to 10 weeks) on a C57BL/6J background were obtained from the Jackson Laboratory. All animal experiments and care procedures were approved and conducted in accordance with the institutional animal care and use committee of the University of Connecticut Health Center (Approval Number: AP-200439-0524). WT and STAT6 KO mice were injected intraperitoneally with folic acid (250 mg/kg, Sigma, St Louis, MO, USA), whereas control mice were administered an equal volume of 0.3 mM NaHCO_3_ [17].

### 2.2. Antibodies and Reagents

Anti-GAPDH was purchased from EMDMillipore (Burlington, MA, USA). Anti-α-SMA (sc-32251, 1:500 dilution) and anti-PDGFRβ (sc-432, 1:500 dilution) were purchased from Santa Cruz Biotechnology (Dallas, TX, USA). Anti-phospho-STAT6 (Tyr641, #9361, 1:1000 dilution) and anti-STAT6 (#9362, 1:1000 dilution) were purchased from Cell Signaling Technology (Danvers, MA, USA). Anti-type I collagen (#1310-01, 1:1000 dilution) and anti-fibronectin (F3648, 1:1000 dilution) were obtained from Southern Biotech (Homewood, AL, USA) and Invitrogen (Carlsbad, CA, USA), respectively. Anti-CD45 (550539, 1:1000 dilution) and anti-CD206 (MCA2235, 1:1000 dilution) were obtained from BD Biosciences (Franklin, NJ, USA) and Bio-Rad Laboratories (Hercules, CA, USA), respectively. The secondary antibodies used were donkey anti-rabbit IgG (HRP, A16035, 1:5000 dilution), donkey anti-goat IgG (HRP, A16005, 1:5000 dilution), donkey anti-mouse IgG (HRP, A16017, 1:5000 dilution), donkey anti-rabbit IgG (Alexa Fluor 488, A-21206, 1:400 dilution), donkey anti-rat IgG (Alexa Fluor 594, A-21209, 1:400 dilution) and donkey anti-goat IgG (Alexa Fluor 488, A-11055, 1:400 dilution) and were obtained from Thermo Fisher Scientific (Waltham, MA, USA). Donkey anti-mouse IgG (Alexa Fluor 488, A-21202, 1:400 dilution) was obtained from Molecular Probes (Waltham, MA, USA).

The Vectastain Elite ABC-HRP kit (Rabbit IgG, PK-6101), Avidin/Biotin blocking kit (SP-2001), antigen unmasking solution (H-3300-250), and DAB substrate (SK-4105) were purchased from Vector Laboratories (Burlingame, CA, USA).

### 2.3. Immunohistochemistry

Kidney tissues fixed in formalin overnight and embedded in paraffin wax were used to detect the level of STAT6 phosphorylation. Sections were subjected to the antigen retrieval method using citrate buffer (Vector Laboratories), followed by the quenching of endogenous peroxidase activity with 3% hydrogen peroxide. Kidney sections were blocked for 1 h followed by incubation with phosphorylated STAT6 antibody overnight at 4 °C. The following day, sections were incubated with biotinylated secondary antibody followed by incubation with ABC solution (Vector Laboratories). Positive staining was detected by incubating kidney sections with 3, 3′ diaminobenzidine tetrahydrochloride (DAB) solution (Vector Laboratories), and hematoxylin was used as the counterstain. Images were captured using a microscope equipped with a digital camera (Nikon, Melville, NY, USA) [22].

### 2.4. Immunofluorescence

Cold acetone was used to fix frozen kidney sections followed by blocking with serum-free protein block. Kidney sections were then incubated with primary antibodies, followed by the corresponding secondary antibodies as previously described [23,24]. Slides were mounted with mounting medium containing 4′,6-diamidino-2-phenylindole (DAPI) (Vector Laboratories). Visualization of fluorescence intensity was assessed using a microscope equipped with a digital camera (Nikon, Melville, NY, USA).

### 2.5. Quantitative Real-Time RT-PCR

TRIzol reagent (Invitrogen) was used to extract RNA from kidney tissues, then RNA was reverse-transcribed, and real-time PCR was carried out using IQ SYBR green assay reagent (Bio-Rad Laboratories). The gene expression was quantified using the comparative Ct method (ΔΔCt) and the relative quantification was subsequently calculated as 2^−ΔΔCt^. In each sample, the target gene expression levels were normalized to GAPDH [9,25]. The primer sequences used in the study are listed below: 

Arg-1 forward, 5′-CTCCAAGCCAAAGTCCTTAGAG-3′, 

-reverse, 5′-AGGAGCTGTCATTAGGGACATC-3′; 

MRC1-forward, 5′-GGTCTATGGAACCACGGATGA-3′, 

-reverse, 5′-TGCCCAGTAAGGAGTACATGG-3′; 

Fizz1-forward, 5′-CCAATCCAGCTAACTATCCCTCC-3′, 

-reverse, 5′-ACCCAGTAGCAGTCATCCCA-3′; 

CCL17-forward, 5′-CGAGAGTGCTGCCTGGATTACT-3′, 

-reverse, 5′-GGTCTGCACAGATGAGCTTGCC-3′; 

GAPDH-forward, 5′-CCAATGTGTCCGTCGCGTGGATCT-3′, 

-reverse, 5′-GTTGAAGTCGCAGGAGACAACC-3′.

### 2.6. Western Blotting

Kidney samples were homogenized with radioimmunoprecipitation assay (RIPA) buffer containing a cocktail of proteinase and phosphatase inhibitors (Fisher Scientific Inc., Rockford, IL, USA). Equal amounts of proteins (50 μg protein/lane) were loaded and separated on SDS-polyacrylamide gels. The separated proteins were then electrophoretically transferred onto nitrocellulose membranes and blocked with a 5% blocking solution. The membranes were incubated with primary antibodies followed by the corresponding HRP-conjugated secondary antibodies. The antibodies used in the study have been validated through prior publications. Chemiluminescence was used to detect the proteins of interest and immunoblots were quantified using NIH ImageJ software [5,26].

### 2.7. Assessment of Renal Fibrosis

Paraffin-embedded kidney sections were used for Sirius red staining to assess collagen fibers [25]. Sections were deparaffinized and rehydrated in ethanol and then stained with Sirius red for 60 min. The stained kidney sections were visualized using a microscope equipped with a digital camera (Nikon, Melville, NY, USA) and collagen fibers were quantitatively evaluated using NIS-Elements Br 3.0 software.

### 2.8. Measurement of Kidney Function

A commercially available kit was used to determine serum creatinine levels (BioAssay Systems, Hayward, CA, USA) as previously described [27,28].

### 2.9. Statistical Analysis

All data are expressed as mean ± SEM. Differences between groups were determined by an analysis of variance (ANOVA) with the Bonferroni procedure for multiple comparison tests. The *p*-value less than 0.05 was considered statistically significant.

## 3. Results

### 3.1. STAT6 Is Activated in the Kidney with Folic Acid Nephropathy

**Figure 1:**
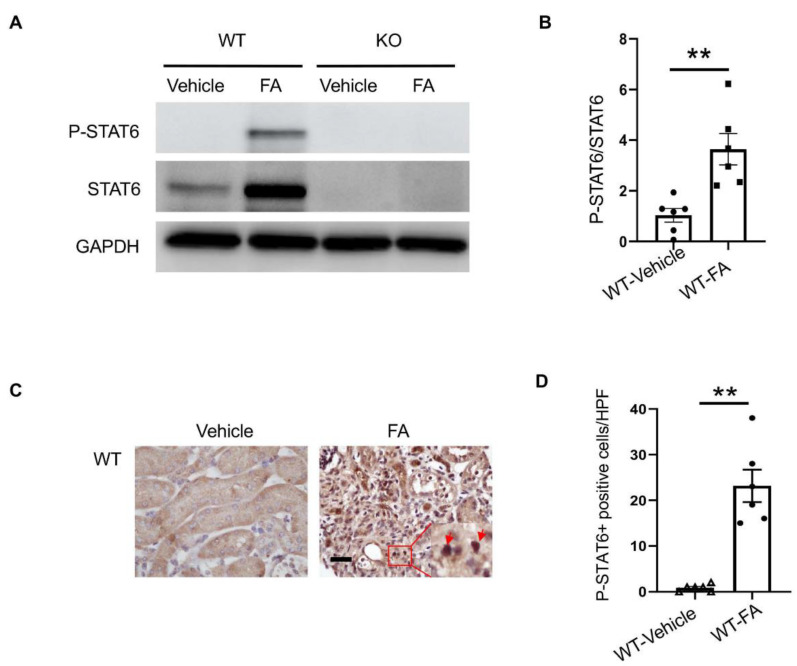
STAT6 is activated in the kidney with folic acid nephropathy. (**A**) Representative Western blots show protein levels of p-STAT6 and STAT6 in the kidneys. (**B**) Quantitative analysis of p-STAT6 in kidneys. ** *p* < 0.01. *n* = 6 per group. (**C**) Representative photomicrographs of kidney sections immunostained for STAT6 phosphorylation (brown) and counterstained with hematoxylin (blue). Scale bar, 50 μm. (**D**) Quantitative analysis of phosphorylated STAT6-positive cells in kidneys. ** *p* < 0.01. *n* = 6 per group.

To examine if STAT6 is activated in the kidney with folic-acid-induced nephropathy, wild-type and STAT6 KO mice were administered vehicle or folic acid. The kidneys were harvested two weeks after administration. Immunoblotting analysis indicated that STAT6 phosphorylation was significantly induced in the kidney following folic acid administration (Figure 1A). Of note, both STAT6 and phospho-STAT6 were not detected in the kidneys of STAT6 KO mice, confirming STAT6 deletion. To determine the types of cells that are responsible for STAT6 activation, kidney sections were immunostained for STAT6 phosphorylation. Phospho-STAT6-positive staining was mainly detected in kidney interstitial cells after folic acid administration (Figure 1B), indicating that STAT6 activation occurs during renal fibrosis development in folic acid nephropathy.

### 3.2. STAT6 Deficiency Impairs Myeloid Fibroblast Accumulation

**Figure 2:**
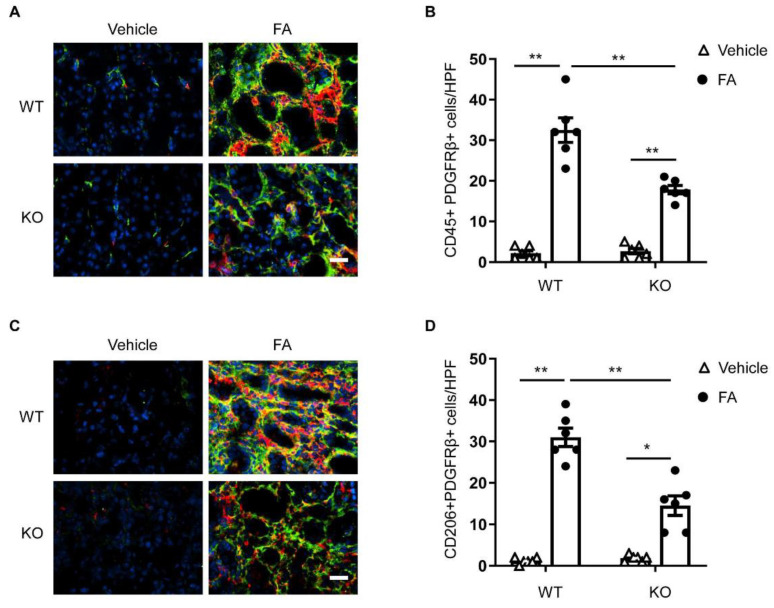
STAT6 deficiency attenuates myeloid fibroblast accumulation and macrophage polarization in folic acid nephropathy. (**A**) Representative photomicrographs of kidney sections from WT and STAT6 KO mice at 2 weeks after vehicle or folic acid treatment immunostained for CD45 (red), PDGFR-β (green), and DAPI (blue). Scale bar, 50 μm. (**B**) Quantitative analysis of PDGFR-β-positive and CD45-positive fibroblasts in kidneys. ** *p* < 0.01. *n* = 6 per group. (**C**) Representative photomicrographs of kidney sections immunostained for CD206 (red), PDGFR-β (green), and DAPI (blue). Scale bar, 50 μm. (**D**) Quantitative analysis of PDGFR-β-positive and CD206-positive fibroblasts in kidneys. ** *p* < 0.01; * *p* < 0.05. *n* = 6 per group.

We then examined whether STAT6 plays a role in myeloid fibroblast accumulation in the kidney with folic acid nephropathy. Kidney sections were immunostained for the leukocyte marker, CD45, and the mesenchymal marker, PDGFR-β. Compared with vehicle-treated mice, mice treated with folic acid accumulated higher amounts of PDGFR-β and CD45 dual-positive cells. The number of PDGFR-β and CD45 dual-positive cells was significantly diminished in the kidneys of STAT6 KO mice with FA treatment (Figure 2A,B). These results demonstrate that STAT6 deficiency attenuates myeloid fibroblast activation in the kidneys of mice treated with FA.

### 3.3. STAT6 Deficiency Attenuates M2 Macrophage Polarization

M2 macrophage polarization has been shown to play a role in the progression of kidney fibrosis [9,12]. A previous study has shown that STAT6 has a key role in the process of macrophage polarization to an M2 phenotype and transformation into fibroblasts [12]. We determined the function of STAT6 in macrophage polarization and myeloid fibroblast transformation in the kidney. Kidney sections were immunostained for an M2 phenotype marker, CD206 and PDGFR-β. An increase in CD206-positive and PDGFR-β-positive cells was observed in the kidney of WT mice in response to folic acid administration. The numbers of CD206^+^ and PDGFR-β^+^ cells were considerably diminished in the kidney of folic-acid-treated STAT6 KO mice compared with folic acid-treated WT mice (Figure 2C,D). These results demonstrate that myeloid fibroblasts arise from monocytes through M2 macrophage polarization, which is driven by STAT6 signaling.

**Figure 3:**
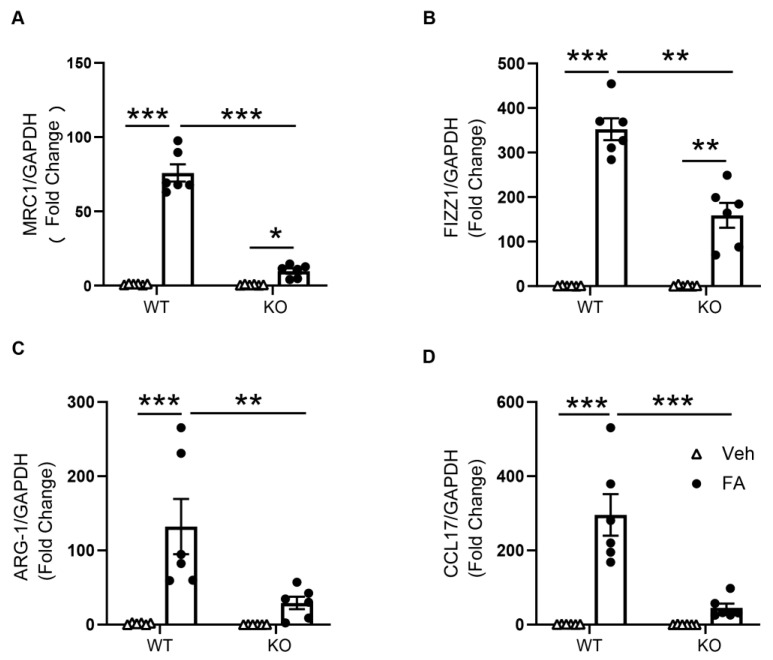
STAT6 deficiency inhibits M2 macrophage markers expression in the kidney. (**A**) Quantitative analysis of MRC1 mRNA expression in the kidneys of WT and STAT6 KO mice. *** *p* < 0.001; * *p* < 0.05. *n* = 6 per group. (**B**) Quantitative analysis of FIZZ1 mRNA expression in the kidneys of WT and STAT6 KO mice. *** *p* < 0.001; ** *p* < 0.01. *n* = 6 per group. (**C**) Quantitative analysis of ARG-1 mRNA expression in the kidneys of WT and STAT6 KO mice. *** *p* < 0.001; ** *p* < 0.01. *n* = 6 per group. (**D**) Quantitative analysis of FIZZ1 mRNA expression in the kidneys of WT and STAT6 KO mice. *** *p* < 0.001. *n* = 6 per group.

The mRNA expressions of M2 macrophage markers were then assessed to further determine the role of STAT6 on M2 macrophage polarization. The mRNA gene expression levels of Arg-1, CCL17, Fizz1, and MRC1 were considerably increased in the kidneys of folic-acid-treated WT mice compared with vehicle-treated WT mice. STAT6 deficiency significantly attenuated the mRNA gene expression levels of Arg-1, CCL17, FIZZ1, and MRC1 in the kidneys with folic acid nephropathy (Figure 3). These results demonstrate that STAT6 signaling drives macrophage polarization to the profibrotic M2 phenotype.

### 3.4. STAT6 Deficiency Inhibits Myofibroblast Transformation

**Figure 4:**
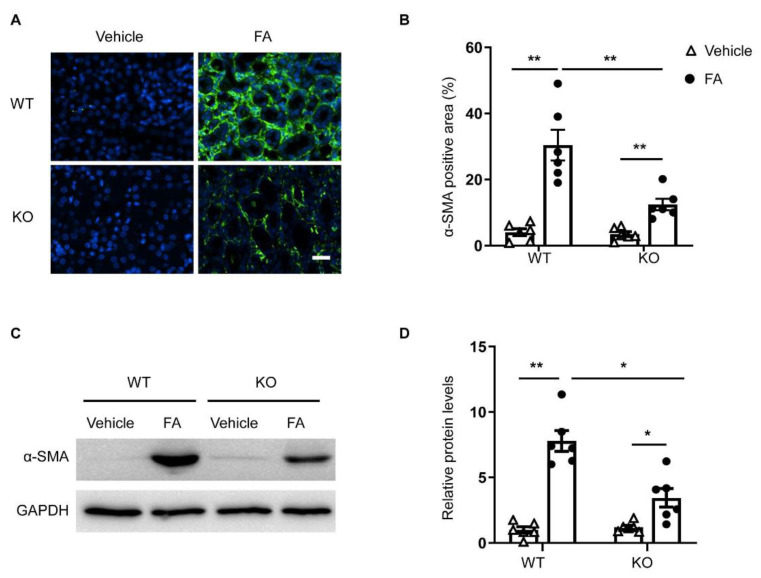
STAT6 deficiency reduces myofibroblast formation in folic acid nephropathy. (**A**) Representative photomicrographs of kidney sections immunostained for α-SMA (green) and counterstained with DAPI (blue). Scale bar, 50 μm. (**B**) Quantitative analysis of the α-SMA-positive area in the kidneys. ** *p* < 0.01. *n* = 6 per group. (**C**) Representative Western blots show α-SMA protein levels in the kidneys. (**D**) Quantitative analysis of α-SMA protein expression in the kidneys. ** *p* < 0.01, * *p* < 0.05. *n* = 6 per group.

To determine whether the deletion of STAT6 affects myofibroblast transformation, kidney sections were immunostained for a myofibroblast marker, α-smooth muscle actin (α-SMA). A greater number of α-SMA positively stained cells was observed in the kidneys of WT mice treated with folic acid. In contrast, genetic deletion of STAT6 significantly diminished the number of α-SMA-positive cells in the kidney following FA treatment (Figure 4A,B). Moreover, the immunoblotting analysis revealed that α-SMA protein level was markedly lower in the kidneys of STAT6 KO mice following folic acid administration compared with folic-acid-treated WT mice (Figure 4C,D). These results indicate that the genetic disruption of STAT6 prevents myofibroblast transformation in folic acid nephropathy.

### 3.5. STAT6 Deficiency Attenuates ECM Protein Production

**Figure 5:**
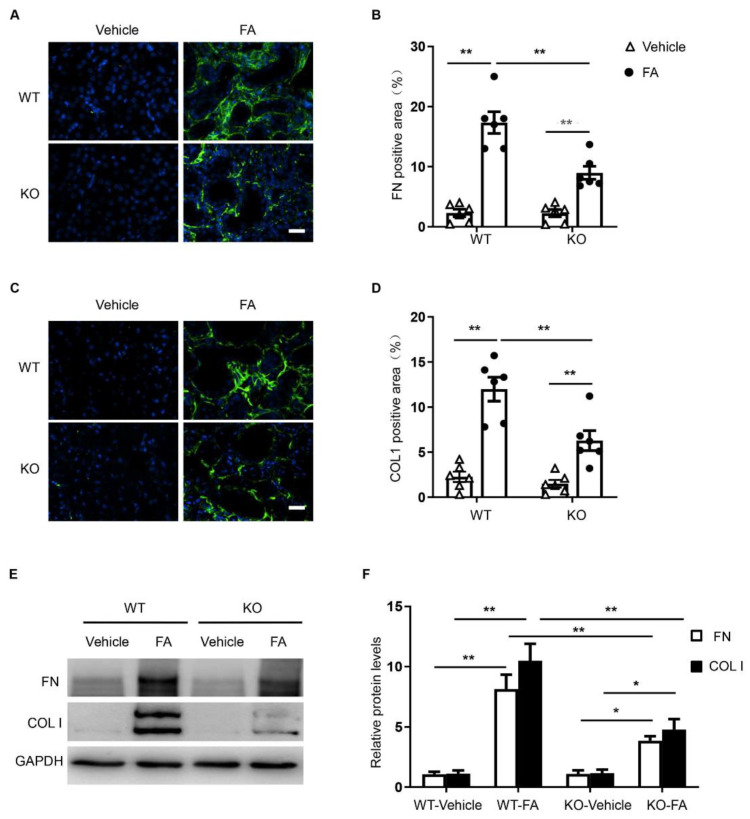
STAT6 deficiency attenuates the expression of ECM proteins in folic acid nephropathy. (**A**) Representative photomicrographs of kidney sections immunostained for fibronectin (green) and counterstained with DAPI (blue). Scale bar, 50 μm. (**B**) Quantitative analysis of the fibronectin-positive area in kidneys. ** *p* < 0.01. *n* = 6 per group. (**C**) Representative photomicrographs of kidney sections immunostained for collagen I (green) and counterstained with DAPI (blue). Scale bar, 50 μm. (**D**) Quantitative analysis of collagen-positive area in kidneys. ** *p* < 0.01. *n* = 6 per group. (**E**) Representative Western blots show protein expression of collagen I and fibronectin in the kidneys. (**F**) Quantitative analysis of protein levels of collagen I and fibronectin in the kidneys. ** *p* < 0.01; * *p* < 0.05. *n* = 6 per group.

To examine the effect of STAT6 deletion on ECM protein production, kidney sections were immunostained for collagen I and fibronectin. The protein levels of collagen I and fibronectin were considerably higher in the kidneys of WT mice treated with folic acid, whereas the genetic deletion of STAT6 significantly reduced collagen I and fibronectin protein expression (Figure 5A,B). Similarly, immunoblotting analysis demonstrates that genetic deletion of STAT6 diminished the increased protein levels of collagen I and fibronectin in the kidneys of mice with folic acid nephropathy (Figure 5C,D). These data demonstrate that STAT6 disruption attenuates ECM production and deposition in the kidney with folic acid nephropathy.

### 3.6. STAT6 Deficiency Ameliorates Renal Fibrosis and Protects Renal Function

**Figure 6:**
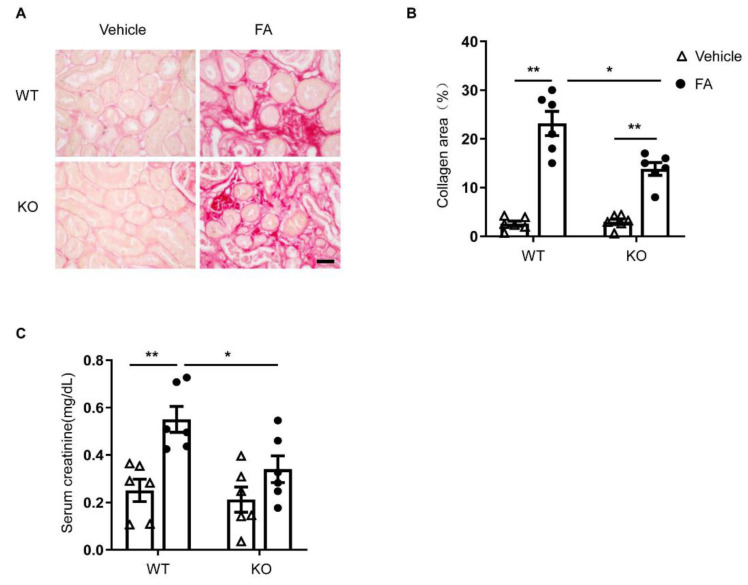
STAT6 deficiency ameliorates renal fibrosis and preserves kidney function in folic acid nephropathy. (**A**) Representative photomicrographs of kidney sections stained with Sirius red for assessment of total collagen deposition in the kidneys. Scale bar, 50 μm. (**B**) Quantitative analysis of interstitial collagen deposition in the kidneys. ** *p* < 0.01; * *p* < 0.05. *n* = 6 per group. (**C**) Effect of STAT6 deficiency on serum creatinine. ** *p* < 0.01; * *p* < 0.05. *n* = 6 per group.

To assess the effect of STAT6 deficiency on kidney fibrosis, kidney sections were stained with Sirius red. Folic-acid-treated WT mice had significant amounts of collagen deposition in the kidney, whereas the collagen deposition in the kidney was markedly lower in folic-acid-treated STAT6 KO mice. These data indicate that STAT6 deficiency ameliorates the pathogenesis of renal fibrosis (Figure 6A,B).

Next, serum creatinine levels were determined to assess the effect of STAT6 deletion on kidney function. WT mice treated with folic acid exhibited significantly elevated levels of serum creatinine (Figure 6C). In contrast, STAT6 KO mice displayed significantly lower serum creatinine levels in folic acid nephropathy. These results demonstrate that deficiency of STAT6 shields the kidney from folic-acid-induced nephropathy.

## 4. Discussion

Kidney fibrosis is a common pathological process in which various CKD progresses to end-stage kidney disease [29,30]. Modern medicine lacks effective drugs for the treatment of CKD and end-stage kidney disease [30]. The main characteristic is that persistent chronic damage causes the kidney’s inherent cells and structures to be constantly destroyed, replaced by the persistent production and accumulation of ECM, and then normal function of the kidney gradually declines [31,32]. The development of effective anti-fibrotic drugs is a long-term goal of nephrologists and scientists in renal medicine around the world. Studies have focused on mechanisms of ECM production during renal fibrosis development. It has been suggested that the transformation of renal intrinsic cells into myofibroblasts is the most important mechanism for ECM production. Compelling evidence indicates that fibroblasts derived from bone marrow recruited into the kidney are an important source of activated fibroblasts responsible for renal fibrosis [9,13,33,34]. We and others have shown that myeloid fibroblasts are recruited into the damaged kidney through chemokines and their respective receptors [35]. Specifically, we have shown that the recruitment of myeloid fibroblasts into the kidney is dependent on the CXCL16 and CXCR6 axes [13,36]. More recently, our studies reported that CXCL16 binding to its receptor CXCR6 activates PI3 kinase γ to recruit myeloid fibroblasts into the kidney, contributing to the development of renal fibrosis [37]. However, how the myeloid fibroblasts are activated into myofibroblasts during the development of renal fibrosis is not fully elucidated.

Inflammatory cells, including T cells, can mediate the activation of bone-marrow-derived fibroblasts. Studies have shown that CD4^+^ T cells can promote the differentiation of peripheral blood mononuclear cells into fibroblasts and play a crucial role in fibrotic diseases [38,39]. CD4^+^ T cells can be differentiated into two subtypes: Th1 and Th2 cells. Th1 cells produce IL-12 and IFN-γ that inhibit fibrocyte differentiation, and Th2 cells secrete profibrotic factors IL-13 and IL-4 that promote fibrocyte differentiation. Studies have found that IL-13 and IL-4 can influence STAT6 activation and play a significant role in immune response [40]. STAT6 is involved in organ and tissue fibrosis, such as kidney and liver [41]. Genetic disruption of STAT6 in tight-skin mice inhibits the development of skin fibrosis [42]. In our previous studies, we have shown that Jak3 inhibition, or the genetic disruption of STAT6, suppresses the accumulation of myeloid fibroblasts and the formation of myofibroblasts in the kidney and inhibits the development of renal fibrosis in mice with obstructive nephropathy [12]. In the current study, STAT6 KO mice were used to determine the role of STAT6 in the activation of myeloid fibroblasts and the development of renal fibrosis in a murine model of folic acid nephropathy. The results demonstrate that the number of PDGFR-β and CD45 dual-positive cells is markedly decreased in the kidneys of STAT6 KO mice compared with WT mice following folic acid treatment. Furthermore, genetic deletion of STAT6 reduces the number of α-SMA-positive cells in the kidney with folic acid nephropathy. These results demonstrate that STAT6 mediates myeloid fibroblast accumulation and myofibroblast transformation in the kidney with folic acid nephropathy.

Macrophages are a group of pluripotent and plastic immune cells that can differentiate into two phenotypes depending on the microenvironments [43]. M1 macrophages promote inflammatory responses by secreting a variety of proinflammatory chemokines and cytokines. In contrast, M2 macrophage polarization is generally driven by Th2 cytokines including IL-13 and IL-4. M2 macrophage polarization has a crucial role in the pathogenesis of organ fibrosis [44,45]. These cell populations are known to secrete a large number of profibrotic cytokines to promote tissue fibrosis [46,47,48,49]. We and others have shown that M2 macrophages mediate renal fibrosis through the transition of monocyte/macrophage to myofibroblast [9,50]. We have also shown that JAK3 inhibitor treatment, or the genetic deletion of STAT6, inhibits M2 macrophage populations and monocyte-to-fibroblast transition, thereby suppressing renal fibrosis in a mouse model of obstructive nephropathy [12]. More recently, we have reported that knockout of IL-4Rα inhibits STAT6 activation and suppresses M2 macrophage polarization and renal fibrosis [17]. In the current study, we demonstrate that a deficiency of STAT6 prevents M2 macrophage polarization and the transition of monocyte-to-fibroblast in folic acid nephropathy. These data suggest that STAT6 mediates M2 macrophage polarization, which has a crucial role in the transition from monocytes to fibroblasts.

Renal fibrosis is the consequence of extensive ECM protein accumulation and deposition, resulting in the disruption of renal parenchyma and progressive decline of renal function. In the current study, we demonstrate that STAT6 deficiency inhibits protein expression of collagen I and fibronectin, two major proteins involved in ECM remodeling in the kidney, and reduces the degree of kidney fibrosis in folic acid nephropathy. Moreover, STAT6 deficiency prevents folic-acid-induced dysfunction in the kidney. These data demonstrate that STAT6 is involved in renal fibrosis and kidney dysfunction in a mouse model of folic-acid-induced nephropathy.

In summary, this study demonstrates that STAT6 signaling has an important role in myeloid fibroblast activation and macrophage polarization during renal fibrosis development in folic acid nephropathy. These findings indicate that targeting STAT6 signaling may provide a novel therapeutic strategy for chronic fibrotic kidney disease.

## Data Availability

The data that support the findings of this study are available from the corresponding author upon reasonable request.
